# Biomaterials and Their Potentialities as Additives in Bitumen Technology: A Review

**DOI:** 10.3390/molecules27248826

**Published:** 2022-12-12

**Authors:** Abraham A. Abe, Cesare Oliviero Rossi, Paolino Caputo

**Affiliations:** Department of Chemistry and Chemical Technologies, Cube 14D, University of Calabria, 87036 Arcavacata di Rende, CS, Italy

**Keywords:** biomaterials, bitumen, bio-oils, polysaccharides, additives, circular economy

## Abstract

The carbon footprint reduction mandate and other eco-friendly policies currently in place are constantly driving the trend of the synthesis and application of sustainable functional materials. The bitumen industry is not an exception to this trend and, every day, new technologies that facilitate safer, cost effective and more sustainable industrial processes and road paving operations are being researched and brought to light. A lot of research is currently ongoing to improve bitumen’s properties due to its use as a binder in road paving processes. Over the years, the most common method to improve bitumen’s properties has been with the use of additives. The major drawback in the use of these additives is the fact that they are substances of strong chemical nature which are either too acidic, too basic or emit toxic fumes and volatile organic compounds into the environment. In the long run, these chemicals are also toxic to the road pavement personnel that carry out the day to day industrial and paving operations. This led researchers to the initiative of synthesizing and applying biomaterials to be used as additives for bitumen. In this light, several studies have investigated the use of substances such as bio-oils, natural waxes, gum, polysaccharides and natural rubber. This literature review is aimed at classifying the different bio-based materials used to improve bitumen’s properties and to provide a deeper knowledge of the application of these biomaterials in bitumen technology. In general, we highlight how the research efforts elaborated herein could potentially foster safer, sustainable, eco-friendly approaches to improving bitumen’s properties while also promoting a circular economy.

## 1. Introduction

Research on biomaterials has been pushed to the forefront of scientific investigation in recent years due to concerns about the emission of greenhouse gases (GHG), global warming, demand for renewable resources and high energy consumption in general. The need for a circular economy and environmental sustainability makes it also important to reduce our dependence on non-renewable resources and drift towards alternative bio-renewable resources for industrial purposes and common day to day activities [1,2,3]. Apart from the economic advantage of the use of bio-derived materials which are less costly and reduce energy expenditure, biomaterials are also environmentally friendly as they are biodegradable, renewable and facilitate strategic resource conservation [4,5,6]. Biomaterials are also known to reduce the greenhouse effect via CO_2_ conservation [7]. All of this goes a long way in promoting a circular economy and eco-sustainability in general. In asphalt technology, biomaterials can be used in different capacities: biomaterials with Warm Mix Asphalt (WMA), biomaterials with Reclaimed Asphalt Pavement (RAP) to rejuvenate aged bitumen, bio-binders as partial or total replacement of petroleum-based binders (bitumen) and so on [8].

The application of bio-binders in the asphalt industry differs depending on the aim of the research study in which it is being used. In the real sense, a bio-binder is a substance of biological origin that serves as an adhesive in the asphalt mix. Conventionally, in the asphalt industry, bitumen functions as the binder but as a result of the continuous research in the field of material science, other more bio-degradable bio-binders are being investigated. The experimental application of bio-binders can be classified, thus [9]:

(a) Direct alternative (75–100% bitumen substitute);
(b) Bitumen extender (10–75% bitumen substitute);
(c) Bitumen modifier (<10% bitumen substitute).

**Figure 1:**
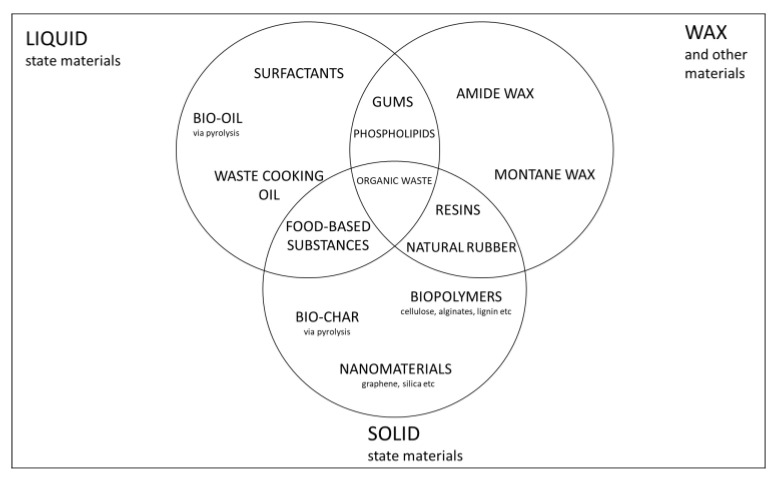
Biomaterials used as bitumen additives and their categorical relativity.

Although the total substitution of conventional asphalt binder is advantageous and preferable, most bio-based binders do not have the necessary properties to produce high quality pavement and for now cannot be used as complete substitutes for asphalt bitumen [10,11,12]. Research is, however, still going on in this regard. This review focuses on the third category listed above i.e., biomaterials as bitumen modifiers (<10% bitumen substitute) because, in existing literature, it is currently the most practically feasible approach to the application of biomaterials to asphalt technology. In addition, studies involving biomaterials as bitumen modifiers are the most abundantly available on scientific database due to the fact that there are more researchers working on this subject [8]. In this literature review, biomaterials and their potential to be used as effective additives to improve bitumen binder’s characteristics in asphalt conglomerate as reported in previous studies will be summarized and discussed. This applies to Warm Mix Asphalt (WMA), Reclaimed Asphalt Pavement (RAP), Cold Mix Asphalt and any asphalt technology which entails bitumen modification in order to produce durable asphalt pavements. Several types of biomaterials have been used in recent years as bitumen additives and quite a number of them have yielded positive results by improving specific characteristics of bitumen. Biomaterials are materials that partially or totally constitute a bio-binder. Biomaterials such as natural waxes, bio-oils, biopolymers, organic waste and bio-based nanomaterials have all been reported in literature to improve bitumen’s characteristics [13,14,15,16,17,18,19]. All the aforementioned types of biomaterials are obtained from different source materials and processes, although, in some cases, different biomaterials can be obtained from the same process or starting material. For example, bio-oil can be obtained via pyrolysis but so can bio-char which can also be classified as a nanomaterial. Bio-char can also be categorized as organic waste but so can Waste cooking oil, which can also be regarded as a bio-based oil. Figure 1 illustrates the classification of the different classes of biomaterials and their relativity to one another.

The different types of biomaterials have different mechanisms of action in bitumen, and this can go a long way in characterizing different classes of biomaterials as additives for bitumen depending on their effect. Bio-oils, for instance, are used as bitumen fluxers by reducing the viscosity of the bitumen and, in the long term, increase the stiffness of the bituminous asphalt pavement by a process called oxidative polymerization. Natural waxes, below their melting point, increase the stiffness and elasticity of bitumen while, above their melting point, decrease the stiffness and increases bitumen’s viscosity. Biopolymers such as polysaccharides and natural rubber modify bitumen by changing its visco-elastic behaviour by increasing bitumen’s range of plasticity. Nanomaterials, on the other hand, often improve the tensile strength of the asphalt conglomerate by forming strong intermolecular crosslinks in bitumen’s matrix, which in the long term translates to increased stiffness and tensile strength of the paved asphalt [9,14,20,21].

From the physico-chemical point of view, a given mechanism of action is the overall result of a delicate equilibrium of all the intermolecular interactions, and therefore dynamic processes, involved: polar and apolar interactions, π-interactions, Van deer Waals and dispersion interactions, eventual H-bonds, etc., all of them exerting their effect with a definite strength and with a definite length-scale and whose combination dictates the complexity of the system [22]. In fact, it is clear that the chemistry of any bitumen is the key element to define the physical properties: following the analogy with reversed micelles in water-in-oil microemulsions, where polar organic domains are stabilized and dispersed in a more apolar matrix, [23,24]. The stabilization of the polar, asphaltene-based domains is of pivotal importance for determining the structure and properties of the overall aggregates, even if the stabilization mechanism must be considered to be quite general, involving, besides organic materials, also inorganic complexes [25], inorganic materials [26] and even nanoparticles [27].

As a consequence of these strict relationships between intermolecular interactions, aggregates structures and their dynamic properties, the rheology (ductility at a given temperature/frequency) and behaviour of bitumen are dependent not only on its structure, but also on the maltene’s glass transition temperature and the effective asphaltene content [28]. Bitumen’s mechanical characteristics, as well as their dependence upon time, ageing, presence of additives and/or modifiers, rejuvenation and so on are therefore interpreted as a result of the tendency of consequent rigidity occurring when stronger intermolecular connectivity appears, compared to a quite less dense intermolecular network of virgin bitumen, where, for example, the asphaltene domains are poorly connected to each other. As a result of the aggregation-based processes of modification, a progressive tuning of the viscoelastic properties can be achieved. In this picture, the resin molecules, due to their amphiphilic nature, have a special role, in our opinion, which needs to be emphasized. In fact, they tend to reduce the associative interactions between the asphaltene particles by interposing itself between the asphaltenes and the maltenes. Similar mechanisms are expected to be common also in bitumen chemistry: the interaction of the apolar part of the resin (its apolar moiety) with the maltene phase will draw the latter towards more hindered dynamics typical of the stiffened asphaltene-dominated structure. Another mechanism can be envisaged, i.e., the formation of direct interactions between the surfactant polar headgroup and polar parts of asphaltene. In fact, it has been recently highlighted that, in addition to polar and apolar interactions, further specific interactions between surfactants themselves with consequent peculiar self-assembly processes [29,30] dictating the final overall aggregation pattern [31] and the (usually slowed-down) dynamics [32,33].

The presence of a wide variety of chemical species and their mutual interactions renders the system certainly complex. This complexity becomes even more marked if we consider that a given molecule in a confined domain can explore different vibrational states giving different populations and therefore different local dynamics. Infra-red studies on model systems [34] have ascertained this possibility and, although not directly influencing the processes studied in this short-review, they must be considered if state-of-the-art techniques for the characterization of the dynamics shown by bituminous molecules are used.

## 2. Bio-Oils

Oils in general are viscous liquids derived from petroleum and are principally used as fuels or lubricants. Due to the diverse nature of oils and the variability of their constituents, for example, fatty acids of differing natures, oils have a broad range of secondary uses and functions. Bio-oils utilize biomass as the starting material instead of petroleum-derivatives. These oils are obtained from the rapid heating of biomass in a vacuum condition [35]. The application of bio-oils in material science is very advantageous because it is renewable, eco-friendly and facilitates conservation of resources by its widespread use [9]. Bio-oils can be obtained by either thermochemical liquefaction or pyrolysis with the latter method being the more eco-sustainable process since it does not involve heavy thermal cracking or hydrogenation of hydrocarbons [36]. Although pyrolysis processes differ slightly in a few parameters and are therefore categorized into, slow, conventional, fast and flash pyrolysis, the commonly used conventional process takes place between 300–600 °C [17,37].

**Figure 2:**
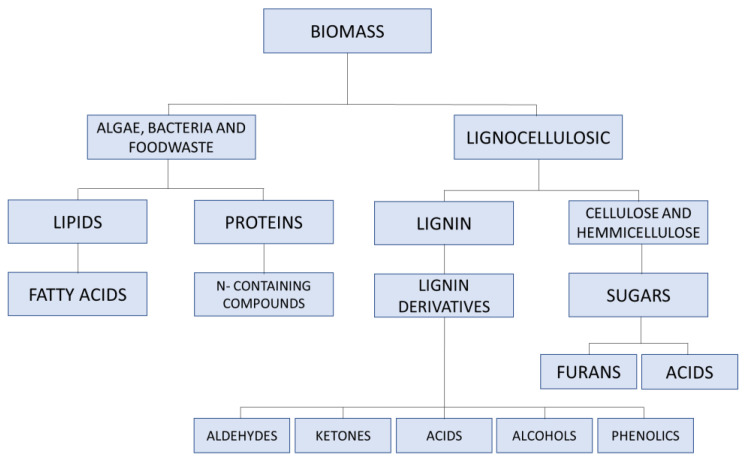
Chemical groups derived from the breakdown of biomass. Reproduced with permission from Machado et al., Bio oils: The next-generation source of chemicals; published by MDPI Reactions, 2022 [40].

Bio-oils from pyrolysis of biomass are liquid emulsions of oxygenated organic compounds, polymers and water. These compounds are mixtures of different components such as furfural, phenols, aldehydes, ketones, ethers, esters and so on. Figure 2 shows the chemical groups of compounds derived from lysis of biomass some of which are described later on in this work. Pyrolysis of biomass also produces another biomaterial as a by-product known as bio-char, which is the solid residue obtained alongside bio-oil. Biochar will also be discussed under a different section of this literature review. Bio oils from pyrolysis are mainly used as bitumen rejuvenators because they contain hydrocarbons which could substitute the hydrocarbons which have been oxidized as a result of bitumen aging [38]. Table 1 shows the composition of pyrolysis bio-oils and the percentages of their components. A 2022 study carried out by Caputo et al. [17] demonstrated that bio-oil derived from pyrolysis has anti-oxidant properties and is capable of regenerating aged bitumen due to the chemical similarities in the hydrocarbons present in the bio-oil and those present in the aged bitumen which have been depleted by oxidative aging. Their study showed that a 2% modification by weight of bitumen with a pyrolysis oil restored the properties of aged bitumen to a state similar to that of unaged virgin bitumen. The carbonaceous nature of the bio-oil’s hydrocarbons brings about an enhanced rejuvenating effect by replenishing the depleted hydrocarbons in the bitumen via adsorption and other chemical interactions which are still under investigation. The effectiveness of pyrolysis oils in rejuvenating bitumen brings about longer-lasting, easy-to-regenerate asphalt pavements thereby promoting resource conservation towards a circular economy. A 2008 study [39] estimated a 23% reduction in energy consumption if asphalt from Reclaimed Asphalt Pavement (RAP) is reused for new paving operations after being rejuvenated by bitumen rejuvenators. In this case, bio-oils derived from pyrolysis can be said to have properties of a bitumen rejuvenator. Not a lot of work has been done on the rejuvenation of RAP bitumen using pyrolysis oils, but it can be projected that, in the next few years, the use of pyrolysis oils in bitumen rejuvenation will be more widespread and researched, thus leading to a better characterization of the exact mechanism of the replenishment of depleted hydrocarbons in aged bitumen.

Apart from pyrolysis-derived bio-oils, several other types of bio-derived vegetable oils exist such as cotton seed oil, hydrogenated palm oil fat, waste cooking oil and hydrogenated palm fat amide. These bio-derived oils from different origins are mainly used to change bitumen binder viscosity in order to reduce asphalt mixture production temperatures, although a few of them might perform more than one function and are more or less multi-functional in their mechanism of action depending on their composition and that of the bitumen as well [14,42,43,44,45]. For example, fatty acid amides present in cotton seed oil were able to rejuvenate oxidized bitumen in Reclaimed Asphalt Pavement (RAP) and increase the fatigue resistance of the asphalt mix when added in the proportion of 7% by weight of bitumen as reported by Nogueira et al. [45]. Hydrogenated palm oil fat and hydrogenated palm fat amide at a 3% by weight of bitumen dosage were also able to rejuvenate oxidized bitumen, modify the mechanical viscoelastic properties and are low-viscosity modifiers of the bitumen tested in the study carried out by Uchoa et al. [42], bio-oil derived from wood residues at 5% by weight of bitumen were found to improve the adhesion of bitumen by 16–21% as reported by Yadykova and Ilyin [44]. Waste cooking oil when used to modify bitumen at a dosage of 2% by weight of bitumen was able to reduce the potential of fatigue cracking of bitumen as reported in a 2018 study conducted by Rasman et al. [43]. Their findings showed that the fatty acids present in the waste cooking oil formed interconnecting structures brought about by oxidative polymerization, thus reducing the fatigue cracking potential of the bituminous mix.

**Table 2:** Fatty acid composition of vegetable oils of different origins (Table adapted from Kamal-Eldin and Andersson [46] with additional data from Asli et al. [47] and Orsavova et al. [48]).

		Fatty Acids (% of Total Fatty Acids)									
Oil Type		Palmitic Acid	Palmitoleic Acid	Stearic Acid	Oleic Acid	Linoleic Acid	γ Linolenic Acid	Lauric Acid	Myristic Acid	Heneicosanoic Acid	Cis-11-Eicosenoic Acid
Waste Cooking	Oil	38.35	<0.3	4.33	43.67	11.39	0.29	0.34	1.03	0.08	0.16
Sunflower		5.2	0.1	3.7	33.7	56.5	0.0	0.02	0.09	<0.4	<0.2
Groundnut		11.2	0.0	3.6	41.1	35.5	0.1	<0.1	<0.1	<0.1	<0.1
Soybean		10.1	0.0	4.3	22.3	53.7	8.1	<0.2	<0.2	<0.2	<0.2
Cottonseed		23.0	0.0	2.3	15.6	55.6	0.3	<0.5	<0.5	<0.5	<0.5
Maize		11.6	0.0	2.5	38.7	44.7	1.4	<0.2	<0.2	<0.2	<0.2
Olive		13.8	1.4	2.8	71.6	9.0	1.0	0.0	0.0	<0.2	<0.2
Palm		44.8	0.0	4.6	38.9	9.5	0.4	0.4	0.5	0.2	0.2
Rapeseed		4.6	0.3	1.7	60.1	21.4	11.4	0.0	0.0	<2.0	<3.0
Linseed		5.6	0.0	3.2	17.7	15.7	57.8	0.0	0.0	0.0	0.0
Sesame		9.6	0.2	6.7	41.1	41.2	0.7	0.0	0.0	<0.3	<0.3
Cashew nut		11.6	0.3	8.9	61.5	17.1	0.0	<0.2	<0.2	<0.2	<0.2
Niger seed		8.8	0.0	6.8	7.5	76.7	0.0	<0.1	<0.1	<0.1	<0.1
Nigella seed		11.4	0.0	2.9	21.9	60.8	0.0	<1.0	<1.0	<1.0	<0.5
Perilla seed		6.4	0.0	1.6	13.8	15.5	62.6	<0.1	<0.1	0.0	0.0

The general chemical mechanism of action of bio-oils in bitumen is explained by a phenomenon known as oxidative polymerization, which brings about crosslinking of the structural units of the polyunsaturated fatty acids which are usually present in oils [14]. Regardless of the initial fluxing and viscosity modifying effect of oils when they are incorporated into bitumen’s matrix, most oils, after solidification and drying of the modified bituminous mix, are reactive to oxypolymerization reactions, which subsequently increase the tensile strength of the asphalt mix. Oils in general contain methyl esters and the precision and effectiveness of the polymerization is dependent on the amount of double bonds present and their position along the aliphatic chain of the fatty acid. Bio-oils vary in their composition and thus have a variable number of double bonds which affects the reactivity of each oil type to oxypolymerization [49].

Fatty acids having at least two double bonds (polyunsaturated) such as linoleic and linolenic acids are abundant in drying oils exposed to air. Monosaturated acids (oleic acid) and saturated acids (stearic and palmitic acids) are also found in oils, but the major determining factor of the reactivity of the polymerization process is the number of polyunsaturated fatty acids [50]. The higher the number of these polyunsaturated fatty acids, the higher the reactivity of the oxypolymerization. The different fatty acids which can be found in different vegetable oils are listed with their respective percentages in Table 2. The crosslinking of the structural units of the fatty acids in the oil via oxypolymerization is brought about by two main reactions. The first is the oxidation of methylene carbon and formation of hydroperoxides from the reaction of radicals with molecular oxygen. The second reaction is the decomposition of the hydroperoxides to alkoxyl radicals. The subsequent crosslinking occurs by radical addition to conjugated double bonds with the formation of higher molecular compounds in the form of ether, alkyl and peroxy bridges [51]. This crosslinking brings about an increase in tensile strength of the paved asphalt. Krol et al. [14] reported that 3.75–5% content of vegetable oils in bituminous mixes is the optimum dosage for effectively improving bitumen binder stiffness and durability.

Biomaterials in solid form such as graphene and silica nanoparticles can also be combined with bio-oils to improve the adhesive properties of bitumen while also conferring tensile strength on the asphalt by the formation of crosslinks facilitated by the presence of the nanoparticles [44,52]. Bio-oils obtained via pyrolysis and even those derived from plant or vegetable origins can also have rejuvenating effects on aged bitumen and reclaimed asphalt. This is because these oils contain esters and heterocyclic rings in their structure. Since asphaltenes are known to be made of condensed aromatic rings and heterocyclic compounds containing nitrogen or sulphur [53], when aged bitumen is modified with bio-oils, the heterocyclic rings contained in the structure of the bio-oils substitute the heterocyclic compounds in the oxidized asphaltenes, thus having a sort of a rejuvenating effect on the aged bitumen. The extent of this rejuvenation depends on the number of heterocyclic rings in the organic compounds of the bio-oil. It is also worth noting that the high temperature performance of bio oil-modified asphalt mixes needs to be extensively investigated subsequently.

## 3. Biopolymers

The term biopolymer has a broad meaning because it refers to a diverse range of materials in different forms. The characteristic that these materials have in common is that they are polymeric substances which are biodegradable regardless of the form they are in (fibres, powders, gums, pellets and so on). Biodegradability of biopolymers depends on their origin and chemical structure in combination with the environmental degrading conditions [54,55]. Depending on the mechanism of degradation, biodegradable polymers are divided into two groups, namely:

(a) Synthetic biopolymers (are degraded via hydrolysis or oxidation);
(b) Natural biopolymers (are degraded enzymatically).

### 3.1. Synthetic Biopolymers

Synthetic biopolymers are mostly biomaterials derived from petroleum sources and are not exactly biopolymers but can be more accurately described as biodegradable polymers. Some are synthetic polymers with hydrolysable backbones, which makes them susceptible to biodegradation under certain conditions. These include polyesters, polyamides, polyurethanes and polyanhydrides [56,57]. The other type of synthetic biodegradable polymers are conventional polymers to which additives have been added to facilitate their degradation. Classic examples of synthetic biopolymers are Polylactic acid, Polyglycolic acid, Polybutylene succinate and Polycaprolactone [58]. Synthetic biopolymers, however, are not commonly used as additives for asphalt and thus are not extensively discussed in this literature review.

### 3.2. Natural Biopolymers

Natural biopolymers are synthesized in biological systems (plants, animals and microorganisms) or are obtained from biological starting materials which have been synthesized artificially—for example, polysaccharides such as starch and cellulose, natural fats and so on. The biodegradability of these biopolymers is very high with the only factor being the varying time frame required for their complete degradation and decomposition, which ranges from a few days to months or even a couple of years [59]. Polysaccharides are a major group of natural biopolymers, with starch and cellulose being the most commonly applied polysaccharides in material science and asphalt technology. Other less common polysaccharides such as alginate and chitosan are also used but to a lesser extent. Natural rubber is another important biopolymer used in asphalt technology and material science in general. Natural rubber is a bio-elastomer which possesses unique characteristics in its pure state or when combined with synthetic elastomers [9,60]. Lignin, phospholipids and animal fat are other naturally biodegradable substances from which biopolymers for asphalt can be obtained.

#### 3.2.1. Polysaccharides

Polysaccharides are one of the most important and investigated natural biomaterials till now. They are also the most abundant class of biopolymers on earth, with cellulose being the most abundant followed by lignin [61]. Polysaccharides are hydrolysable macromolecules made up of several monosaccharide units bound together via glycosidic linkages. The flexibility, low cost, biological importance and wide range of application of polysaccharides as biomaterials makes them a force to reckon with in research, and this is reiterated by the constant interest of researchers in this particular class of biomaterials. In recent years, new methods to modify and customize polysaccharides, their biodegradability and ability to synthesize certain structures have definitely increased the desirability of the application of polysaccharides as functional sustainable materials [56]. In the context of asphalt, polysaccharides are used as eco-friendly additives due to their ability to form structural units depending on the conditions they are subjected to. They have a wide range of applications as they can be used to improve bitumen’s properties in Warm Mix Asphalt (WMA), bituminous emulsions and even in Reclaimed Asphalt Pavement (RAP) rejuvenation [15,16,62]. In WMA, they exhibit a sort of structural crosslinking in bitumen’s matrix, which increases its rigidity and temperature resistance. Polysaccharides derived from algae have been reported to have potential rejuvenating effects on aged bitumen present in Reclaimed Asphalt Pavement (RAP) and have also been reported to improve homogeneity in bituminous emulsions. A few natural fibres in the form of lignocellulosic complexes are polysaccharide-based. Natural fibres in general are discussed later on in this review. A few polysaccharides existing in the form of natural gums have also been shown to be effective in improving bitumen’s rheological properties [62].

##### Natural Fibres

Natural fibres are biomaterials obtained from the bodies of plants or animals and their major application is their use as components of composite materials. Fibres are used in composite systems because they impart their fibrous properties into the structure of the composite often conferring a reinforcing effect [63,64]. This is the concept behind the use of natural fibres as additives in bituminous systems. The efficiency of natural fibres such as cellulose fibres, lignin fibres, sisal fibres, coconut fibres and jute fibres as rheological modifiers and reinforcing agents in bitumen has been a subject of investigation in several research studies [65,66,67,68,69,70,71]. Some natural fibres contain a mixture of different fibrous materials in order to obtain fibres of higher quality with desirable physicochemical properties. For instance, natural cellulose fibres are composed of cellulose microfibrils in a matrix of hemicellulose and lignin [72] as shown by the different types of biomass byproducts represented in Figure 2. In conventional natural cellulose fibres, hemicellulose is responsible for the moisture absorption, biodegradation and thermal degradation while lignin ensures thermal stability but is responsible for UV degradation [73,74]. In asphalt, cellulose is used as a reinforcer to increase the tensile strength of the bituminous mix by modifying its rheological properties. Data from literature demonstrate that different types of cellulose fibre such as jute, cotton, hemp, sisal and coconut fibres improve the fatigue performance, tensile stress and stiffness of asphalt conglomerate. Paramita [69] suggests that cellulose fibres containing higher cellulose and lignin content provide extra water resistance and stability to the asphalt mix. It has also been proven that microfibrillated cellulose decreases the temperature sensitivity of bitumen both in low and high temperature ranges [18]. A 2016 study demonstrates that cellulose-based fibre significantly prevents rutting phenomena of asphalt at high service temperature [70].

The varying percentages of the components of the different types of cellulose fibre bring about slightly different results in asphalt mixes depending on the respective composition of the fibres. Kumar et al. reported that stone mastic asphalt (SMA) containing 0.3% jute fibres by weight of mixture showed improved bitumen adhesion and the substitution of synthetic fibres with natural jute fibres brings about an 18% reduction in construction cost per metric ton of the SMA mix [75]. Coconut and hemp fibres at a dosage of 0.3–0.4% by weight of bitumen reduce rutting phenomena and improve fatigue life of asphalt but have no effect on cracking resistance [76,77]. A 2019 study conducted by Kundal and Goel [66] demonstrated that sisal fibre at a dosage of 0.4% by weight of mix increases the stability and durability of bituminous mixes while decreasing air void and flow values. Oyedepo et al. [67] reported an increase in the stability of bituminous mixes modified with different dosages of sisal fibre ranging from 0.1–0.5% by weight of bitumen. The highest stability value was obtained at the 0.2% dosage. This dosage also gave the highest flow values indicating the reinforcing effect of the fibres, thus improving the resistance of the bituminous mix to plastic deformation (flow). They also reported, however, that a greater compaction force is required for sisal fibre reinforced asphalt conglomerate. A few studies have shown that lignin fibre improves fatigue performance, water stability and resistance to low temperature cracking in asphalt mixes [78,79].

##### Chitosan

Chitosan is a linear polysaccharide synthesized from chitin which is found in the exoskeleton or shells of arthropods and crustaceans such as insects, shrimp and crabs. It is an FDA approved food additive and is also one of the most abundant natural biopolymers because it is actually considered as amino-cellulose [55,56]. The degradation of chitosan occurs through the hydrolysis of its acetylated residues and the rate of this degradation inversely depends on the crystallinity and the degree of acetylation of the polymer [80]. Chitosan can be morphed into various forms and structures such as nanofibres, nanospheres and gels due to its structural flexibility. This morpho-structural flexibility coupled with its outstanding biocompatibility, pH sensitivity and excellent biodegradability makes chitosan ideal for applications in material science and engineering [56].

The main application of chitosan in bitumen technology is as an additive to bituminous emulsions. In the study carried out by Mallawarachchi et al. on bituminous emulsions [16], protonated chitosan was used to partially replace the emulsifier in the proportion of 10–20% (*w*/*w*) of chitosan. The chitosan–emulsifier mixture was then used in the production of the bituminous emulsion at a dosage of 0.23–0.9%. Their findings demonstrated that protonated chitosan was able to stabilize cationic bituminous emulsions by improving the stability and viscosity by forming interactions between the negatively charged atoms in the bitumen’s asphaltenes and the positively charged amine groups in chitosan. Conventional chitosan on its own is not a great emulsifier, and it needs to be subjected to low pH conditions in order for protonation of the reactive amino groups along chitosan’s backbone to occur [81]. Chitosan stabilizes the emulsion by dissolving in the aqueous phase and dispersing the oil (in this case bitumen) by increasing the matrix viscosity. This is brought about by the formation of dense polyelectrolytic brush on the water side of this oil–water interface. This polyelectrolytic brush is formed by the assembly of chitosan on the oil–water interface with a few hydrophobic anchoring points which produces a dense brush of hydrophobic loops and chain ends on the water layer and a relatively small effect on the oil layer [82]. The best cationic emulsifying agents for bituminous emulsions are those which possess a hydrophobic tail and lipophilic head group of nitrogenous compounds [16] and the protonation of chitosan’s reactive amino groups which activates their ability to generate cationic type surfactants brings chitosan into this category of ideal emulsifiers for cationic bituminous emulsions. Another study carried out by Chapelle et al. [83] demonstrates that functionalized chitosan in the form of polymerized chitooligosaccharides modified with different lipophilic chains were able to stabilize oil in water emulsions when applied at a dosage of 1% (*w*/*w*). In their study, the chitosan-based biosurfactant polymerized to a degree of 10 proved to possess interesting interfacial properties coupled with its ideal particle size, which directly influences the stability of oil in water bituminous emulsions. Chitosan can be a bio-based solution to the problem of the use of toxic chemical bitumen emulsifiers in the asphalt industry.

##### Starch

Starch is one of the most widely used polysaccharides and its applications cut across many fields. It is a hydrocolloid biopolymer which is very cheap, abundantly available and highly biodegradable. Hydrocolloids are capable of forming gels or providing viscous dispersions in the presence of water [84]. Starch is a combination of two types of glucose: amylose which is a linear, crystalline polymer and amylopectin, which is a branched, amorphous polymer [85]. The ratio of amylose to amylopectin has a huge impact on the physicochemical properties of starch, and this ratio varies depending on the starch source [86]. The higher the amylose content, the greater the elasticity and strength of the materials derived from the starch [55,87,88]. Starch can be used in its granular, powdery form as a filler in resins and colloidal systems or it can be melted to obtain thermoplastic starch which can be used in starch-based biocomposites [89]. In order to realize thermoplastic starch, the starch needs to be destructured by treating it with polyols, water and heat. The polyols trigger a recrystallization reaction known as retrogradation [90,91]. In order to improve the mechanical properties of starch, it can be acetylated. Starch acetate has a higher amylose content and is more hydrophobic, thus having improved mechanical properties compared to unmodified starch [92].

In asphalt, starch has been proven to reinforce bituminous mixes in several ways. Porto et al. [62] reported an increase in rigidity and resistance to high temperatures of bitumen when modified with starch at a dosage of 4.8% (*w*/*w*). This was brought about by the high number of polar groups in the tested starches which were able to bind to the polar clusters of asphaltenes and their super assembled aggregates thus forming an interconnected network. These findings were corroborated by Iwaura and Komba [93] in their study which demonstrated that starch self-assembled into a supramolecular lamellar fibre network with bitumen’s matrix, thus increasing its viscosity at high temperatures. Their study also demonstrated that starch at dosages of 5, 10 and 16% (*w*/*w*) improves the rheological properties of bitumen and increases its elasticity in the temperature range of 20–60 °C. They showed that starch was able to increase bitumen’s viscoelasticity by forming a three-dimensional fibre network structure by forming noncovalent interactions with both the asphaltenic and maltenic fractions of bitumen. Their findings suggested that starch-modified asphalt can be produced at lower temperatures, in less time and with simpler methods compared to conventional polymer-modified asphalt pavements. A 2010 study by Al-Hadidy et al. [94] showed that a 5% starch-modified asphalt had reduced temperature susceptibility at high temperatures, rutting potential and moisture susceptibility with an increased shear resistance. The increased moisture resistance of the starch-modified asphalt makes the starch additive a good anti-stripping agent. Their results also revealed that the starch–asphalt binder was resistant to most common chemicals and fuels, making it an ideal paving option for strategic places such as fuel stations. A recent study by Jack and Iwo reported that corn starch in a proportion of 5% by weight of asphalt improved the strain resistance, stiffness and fatigue performance of asphalt concrete, emphasizing the capacity of starch as a bitumen reinforcer [95]. Some of the other general findings of the studies [93,94] suggest that starch is one of the most eco-friendly bitumen additives due to its high degradability, low cost and ability to reduce emissions and energy costs by lowering production temperatures.

##### Alginates

Alginate is a polysaccharide extracted from brown algae via a base solution. Alginic acid is formed when alginates are reacted with acid and the alginic acid produced is capable of gelling in the presence of divalent cations (counterions) such as Ca^2+^. This gelling tendency enables the encapsulation of various molecules within alginate gels [55]. Conditions such as pH and counterion type affect the gelling extent and degree of structural crosslinking in alginate gels [96].

The ability of alginate to form gels and encapsulate various components is the underlying mechanism behind the application of alginates in bitumen and asphalt technology in general. Alginate on its own or in combination with crosslinking agents and/or rejuvenators have been shown in a few studies to be capable of rejuvenating aged bitumen, thereby increasing the life cycle of asphalt pavements facilitating the reuse of Reclaimed Asphalt Pavement (RAP) in new paving operations. Xu et al. [97] demonstrated that calcium alginate capsules at a dosage of 8% by weight of bitumen induce a self-healing property in porous asphalt mixes by facilitating crack healing in the asphalt mastic. They administered calcium alginate in combination with a rejuvenator into the asphalt mix. The calcium alginate capsules were able to form gels within the asphalt mix, encapsulate the rejuvenator and later release the encapsulated rejuvenator at crack sites within the asphalt mastic, thus propagating a sort of self-healing effect. The calcium alginate was also able to reinforce the asphalt mix by improving its stiffness modulus. These results corroborated the findings of an earlier study [98] conducted by the same researchers in 2018 in which calcium alginate capsules were demonstrated to be an effective method for the encapsulation and delivery of rejuvenator to damage sites with the asphalt mastic mix.

Malinowski et al. [15] showed that sodium alginate at a dosage of 2.5% (*w*/*w*) combined with epichlorohydrin as a crosslinking agent had a softening effect on aged bitumen due to the crosslinking of the biopolymer in the bitumen binder volume. Higher molecular weight structures are formed during this crosslinking and are positioned between asphaltene clusters, thus limiting asphaltene aggregation and reducing binder hardening. The chemical reactions of the -OH groups of the sodium alginate and the oxygen atoms present in the aged bitumen structure were also reported to be partially responsible for the softening effect observed. In the asphalt mix, the sodium alginate also brought about improved aggregate coating, better workability, improved compactibility and reduced air void content. The most important finding of their research study, however, was the significantly enhanced water and frost resistance of the asphalt mix modified by sodium alginate. Porto et al. [62] also reported that alginate extracted from *Eucheuma* spp. applied at a dosage of 4.8% (*w*/*w*) improves the thermal resistance of bitumen and especially improves the rheological properties of bitumen by establishing soft bridges which connect the different asphaltene aggregates and strengthen their overall hierarchical supra-structures.

#### 3.2.2. Natural Rubber

**Figure 3:**
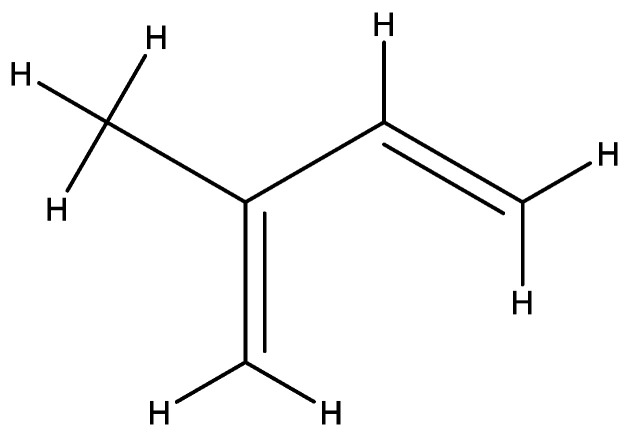
Chemical structure of an Isoprene molecule.

Natural rubber (NR) is a bioelastomer which is obtained from the milky latex (sap) of the tree species *Hevea brasiliensis.* Natural rubber in its pure form consists of the organic compound polyisoprene, water, resins and some impurities. Natural rubber occurs in the form of sap, a sticky, milky-like liquid which is a colloidal dispersion of polyisoprene molecules suspended in aqueous medium [99,100]. NR is a widely available, multipurpose substance which is used in the production of several commodities such as tires, balloons, gloves and mattresses. Its thermal and binding properties have brought about its use in improving the properties of bitumen binder in asphalt pavements [101,102]. Due to double bonds which are present in the repeated units of natural rubber, it can easily undergo crosslinking or vulcanization, a property which is desired in asphalt mixes; however, in order to possess stable elasticity, rubber products must exhibit three-dimensionally structured networks [103]. This is the idea behind the incorporation of natural rubber in asphalt mixes. Natural rubber’s elastic properties are due to its high stretch ratio and resilience as a result of its flexible polyisoprene chain containing an amorphous mass of coiled structures. This polyisoprene chain behaves like a linear chain when a load or stress is applied and then wrinkles and reverts back once the load is removed [104]. The chemical structure of isoprene is shown in Figure 3. Unprocessed NR is not ideal for direct incorporation into bitumen as an additive due to its high water content. In order to prevent bacterial attack on natural rubber latex, small amounts of ammonia are usually added to it [105,106]. However, high concentrations of solid rubber can be obtained by centrifugation, evaporation and creaming of the raw latex [104,107].

Natural rubber which is used to modify bitumen exists in different forms: natural rubber latex, liquid natural rubber, ribbed smoked sheet and natural rubber powder. Liquid natural rubber is the most preferred among the aforementioned forms of natural rubber because it blends better with bitumen and produces a more homogenous mix [108,109]. In asphalt, natural rubber is an ideal bioelastomer because of its beneficial properties such as elasticity, high tensile strength, crack growth resistance and flexibility. Its viscoelastic behaviour implies that, before it undergoes crosslinking, it exists in the form of a viscous liquid and, after crosslinking, it solidifies and exhibits high elasticity [110,111]. Another beneficial property of natural rubber is its ability to crystallize when cooled at low temperatures or by applying strain in a particular direction. Crystallization in cooled rubber limits the movement between neighbouring molecular chains, thus leading to a substantial increase in tensile strength [112]. This strain-induced crystallization makes so many industries and sectors rely heavily on NR because it confers resilience, tensile strength, elasticity, low heat build-up and abrasion on the resulting product [99]. In cold weather, natural rubber has been shown to prevent crack formation while maintaining bitumen stiffness by forming a sort of elastic band in the bituminous mix. At higher temperatures, natural rubber acts as a film which improves shear resistance, which, in turn, increases bitumen’s viscosity [106,113]. NR has also been reported to improve the cost-effectiveness of road paving operations due to its low cost while also increasing the service life of the asphalt pavements even with little or no maintenance carried out [114]. Krishnapriya in a 2015 study [115] demonstrated that bituminous mixes modified with 2% NR by weight of bitumen exhibit excellent rut resistance and improved resilient modulus and fatigue life. In the same year, Shaffie et al. reported in their study that bituminous mixes containing 8% NR by weight of bitumen exhibited a better resistance to stripping phenomenon [116]. It has been generally proven that NR with its fatigue resistance, good tear strength and higher stability is able to extend the stability and long-term performance of asphalt pavements [116,117]. NR also presents an economical advantage due to its low cost and the practicality in some of its different forms; however, for some of its applications in bituminous mixes, it may have to be slightly modified to preserve the natural rubber latex itself [105,106,109].

## 4. Waxes

Waxes are lipophilic organic compounds such as lipids and higher alkanes which are insoluble in water but soluble in organic, non-polar solvents. They consist of long chains of aliphatic alkyls and sometimes even aromatic compounds. Depending on the source of the wax, they also contain various functional groups such as fatty acids, aldehydes, alcohols and esters of fatty acids [118]. Waxes have a number of industrial applications but are mostly combined with other substances to produce coating formulations and colourants [119]. Industrially, synthetic waxes are more widely used, but, recently, due to the need for eco-friendly practices, the demand for the use of natural waxes is increasing. This is due to the biodegradability of natural waxes, which the synthetic waxes such as paraffin wax are not capable of.

In bitumen technology, waxes and wax-based additives are used to lower bitumen viscosity, improve workability and enhance lubrication [120]. The reduced viscosity brings about an increased stiffness, which occurs as a result of the solidification of the wax into microscopic particles uniformly distributed in bitumen’s matrix upon its cooling [121]. Waxes have melting points below asphalt processing temperatures and thus melt and become dispersible in the mix during asphalt production processes. Since waxes are apolar, they feed the maltene fraction of bitumen, thus improving its flow and workability during asphalt paving processes, and this would better disperse asphaltene clusters at their various levels of aggregation in bitumen’s matrix [122,123]. In most cases, waxes have little or no effect on bitumen’s rheological properties even though they modify the binder’s viscosity. Caputo et al. [124] reported that a waste food wax additive at dosages of 0.4–3% by weight of bitumen improved the workability of asphalt conglomerate acting on the viscosity of the bitumen without changing its rheological properties. Sigwarth et al. in a recent study [20] tested the efficiency of 10 bio-degradable waxes and their findings show that the effects of the waxes on bituminous mixes were significantly affected by the wax origin, composition and specific melting point. The waxes when applied in a 3% proportion by weight of bitumen were able to increase the stiffness and elasticity of the bitumen above their melting point temperatures. The results also demonstrated that two of the waxes performed excellently and have the potential to completely replace the synthetic waxes (Sasobit and Licomont), which were compared to the biodegradable waxes in the study. Oliviero-Rossi et al. [13] tested the potential of a food-grade phospholipid wax as a bitumen additive, and their results show that the phospholipid at dosages ranging from 1–6% improved bitumen’s rheological properties. They also demonstrated that the phospholipid wax enhanced the adhesion between bitumen and the aggregates in asphalt mix by reducing the interfacial energy between the aggregates and bitumen binder. In general, the applicability of natural waxes as bitumen additives, apart from being eco-friendly, can also foster circular economy via recycling, environmental protection by reducing harmful emissions during production processes and resource conservation since most of these wax-based additives are waste products of other processes and are, most importantly, biodegradable.

## 5. Nanomaterials

The use of nanomaterials is becoming increasingly popular in the asphalt industry due to the fact that nanotechnology principles can be applied to modify the characteristics of materials at the atomic (nano) level. Macro-properties of materials can be modified at the nano scale. At the nano scale, the material properties are noticeably different compared to the macro scale. This is due to higher surface area to volume ratio at the nano scale and, at such small dimensions, quantum effects are pronounced [125]. In general, nanomaterials have particle sizes between 1–100 nm, and this allows for the formation of nanostructures within bitumen’s matrix, thereby conferring some beneficial effects on the bitumen of interest [126]. Most nanomaterials used in asphalt improvement are of natural origins with a huge percentage of these materials being mineral substances which are not biodegradable in the real sense. Some of these mineral-based nanomaterials include nanoclay, graphite/graphene and their oxides, nanosilica and carbon nanotubes. Biochar, which is a by-product of pyrolysis of biomass, is also a nanomaterial of interest and can improve the properties of bituminous mixes. A few food-based powder additives are also capable of forming nanostructures within bitumen.

### 5.1. Biochar

Biochar is a carbonaceous black residue, which is a by-product of the thermal degradation (pyrolysis) of biomass. In bitumen, it is usually applied in its powder form, and it has been shown to improve bituminous mixes in different ways. Several studies have characterized the physicochemical improvements of bitumen modified with biochar and the mechanical improvements of asphalt obtained from these char-modified mixes [17,127,128,129]. Caputo et al. [17] demonstrated that bio-char obtained from the pyrolysis of municipal solid waste when applied in a proportion of 2% (*w*/*w*) in bituminous mixes improve the mechanical properties of bitumen as evidenced by the rheological results in their study. Their findings also indicate that bio-char has a high potential as an effective filler for bituminous conglomerates. A study carried out by Zhao et al. [128] demonstrated that bio-char obtained from the pyrolysis of switchgrass biomass was more effective in improving bitumen’s rheological properties and temperature susceptibility than commercially available active carbon. Their results showed that using bio-char as a bitumen modifier at a dosage range of 5–10% increased the viscosity of the asphalt binder at high service temperatures while showing little effect at low service temperatures. The bio-char investigated in their study also reduced the effect of long-term oxidative aging of the asphalt binder to some extent. The rutting resistance of the asphalt binder at high service temperature was also significantly increased due to the effect of bio-char. Gupta et al. [129] also reported that two biochars obtained from the pyrolysis of food waste and mixed wood waste respectively when incorporated in the range of 1–2% (*w*/*w*) into asphalt and cement mortar mix reduced water penetration by 40%. The mixed wood waste biochar improved the tensile strength of the mortar mix by 20%. In general, both biochars proved to be effective reinforcers of the pavement mortar mix. Biochar’s nano-sized particles even when added in small percentages can effectively modify bitumen’s rheological properties due to their high surface to volume ratio and customizable chemical composition depending on the biomass starting material [130]. Biochars having low particle size values have been shown to give better results as the nanostructures formed by these biochars decrease the formation of cracks and increase the load capacity of the asphalt pavements. The higher the carbon content of the biochar, the higher the compatibility with bitumen since they are both carbon-rich materials [131,132,133]. A few studies have also demonstrated that biochar has anti-aging effects on bitumen due to the interaction of the apolar residues of biochar with the maltenic fraction of bitumen restricting the dynamics of the maltenes, which in turn could slow down the process involving the dynamics of asphaltene clustering and aggregation [134,135]. These aforementioned studies indicate that the effectiveness of biochars in improving bitumen’s properties promotes waste recycling and re-utilization, thus preventing improper disposal and pollution of the environment while improving civil infrastructure.

### 5.2. Nanoclay

Nanoclays are nanoparticles of thin sheets of two-dimensional layered mineral silicates which are about 1 nm thick and about 70–150 nm wide. They are also relatively cheap compared to polymer-modified binder since they occur naturally. Nanoclays are categorized depending on the morphology of their nanoparticles and their chemical composition. These categories include montmorillonite, kaolinite, bentonite and hectorite. Montmorillonite is the most widely used form of nanoclay, and it is composed of multi-layered stacks of aluminosilicate with metal cations on its surface. The efficiency of nanoclays in improving bituminous mixes depends on factors like particle size distribution, treatment type, organic modification and specific surface area [126]. Nanoclays as additives in bitumen have been shown generally to increase Marshall stability, stiffness modulus at high temperature, fatigue resistance and moisture damage resistance while lowering permanent deformation when used to modify bitumen at dosages ranging from 1–7% [126,136].

### 5.3. Graphite, Graphene and Their Oxides

**Figure 4:**
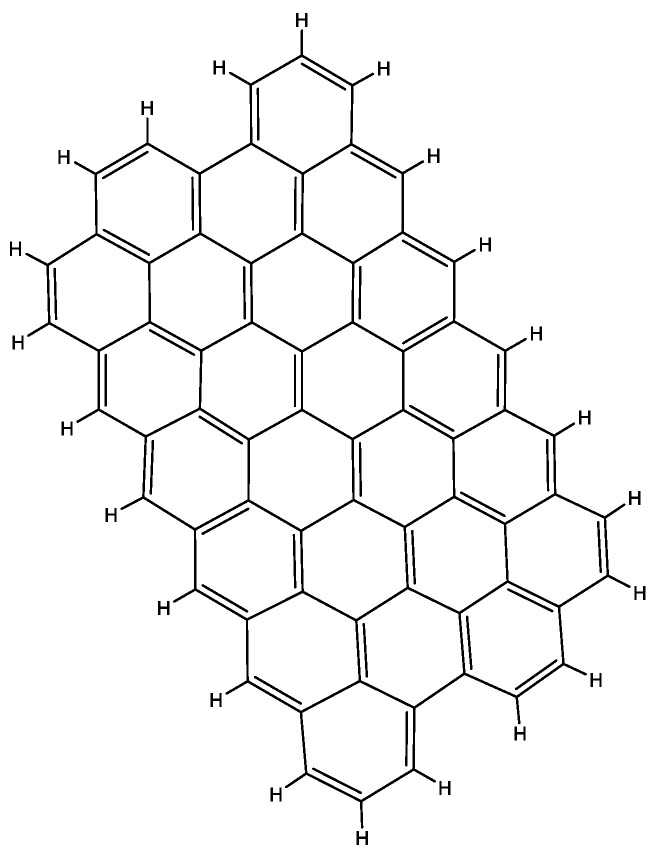
Structural representation of a graphene sheet.

Graphene is an allotrope of carbon whose structure is an arrangement of a layer of atoms in a honeycomb lattice nanostructure. Other carbon allotropes such as diamond are known for their lattice structures which make them some of the hardest materials on earth, and graphene is not an exception [137]. Graphene and graphene oxide are two of the most promising materials in construction in general because of their exceptional electrical and physical properties [138]. Graphene which is simply one atomic layer of graphite has very interesting and peculiar properties, which make it ideal as a bitumen modifier. It is a very hard, difficult to break to surprisingly flexible material. This flexibility is due to the fact that it occurs in sheets. This sheeting dispersion can be seen in the distribution of graphene molecules in the chemical structure shown in Figure 4. Graphene oxide has been shown to enhance aging resistance of asphalt mixes, weaken the influence of aging on the low temperature performance of these mixtures and also reduce moisture susceptibility of aged mixtures [126,139]. Asphalt mixes modified with graphene oxide have shown a significant resistance to cracking damage and rutting phenomena [140]. Researchers from the University of Minnesota reported that asphalt mixes modified with graphite nanoplatelets (GNPs) exhibit significantly improved mechanical properties at low temperatures. They reported that GNP-modified asphalt binders show greater flexural strength at low temperatures compared to conventional asphalt binders. According to their findings, incorporation of GNPs into bituminous mixes in the proportion of 3–6% by weight of binder can improve flexural strength by 130%. They also demonstrated that GNPs improve the rutting performance of asphalt mixtures and increase the indirect tensile strength and fracture energy of the mixtures. These investigators attributed some of the improvement of the asphalt mixtures to the fact that GNPs are very easy to disperse in asphalt binders. Due to their relatively low aspect ratio, GNPs bring about no clustering at all during mixing processes [52]. The application of graphene as an asphalt additive is quite new and has only recently begun to be exploited so it still needs to be extensively investigated. However, on the market, graphene-based additives for asphalt exist such as GiPave by Italian manufacturer, Iterchimica.

### 5.4. Nanosilica

Nanosilica is obtained from micro-based silica, and it has a particle size of about 10–15 nm. Silica is a naturally occurring form of silicon dioxide [141]. Nanosilica is used as a bitumen additive due to its excellent properties such as its large surface area, excellent dispersion capacity, strong adsorption and high chemical purity. A few studies have used nanosilica as nanomodifiers, and it was shown that Warm Mix Asphalt modified with nanosilica particles exhibited reduced cracking depth, improved fatigue life and reduced rutting depth [142,143]. Mahmoud and Aboelkaim modified bitumen with naosilica at 2, 4 and 6%, and their findings show that nanosilica increased the softening point and viscosity of the bituminous mix while decreasing the penetration grade value. It is also worth noting that further infrared analysis carried out in the same study revealed the presence of multiple functional groups in the modified bitumen [144].

### 5.5. Carbon Nanotubes

**Figure 5:**
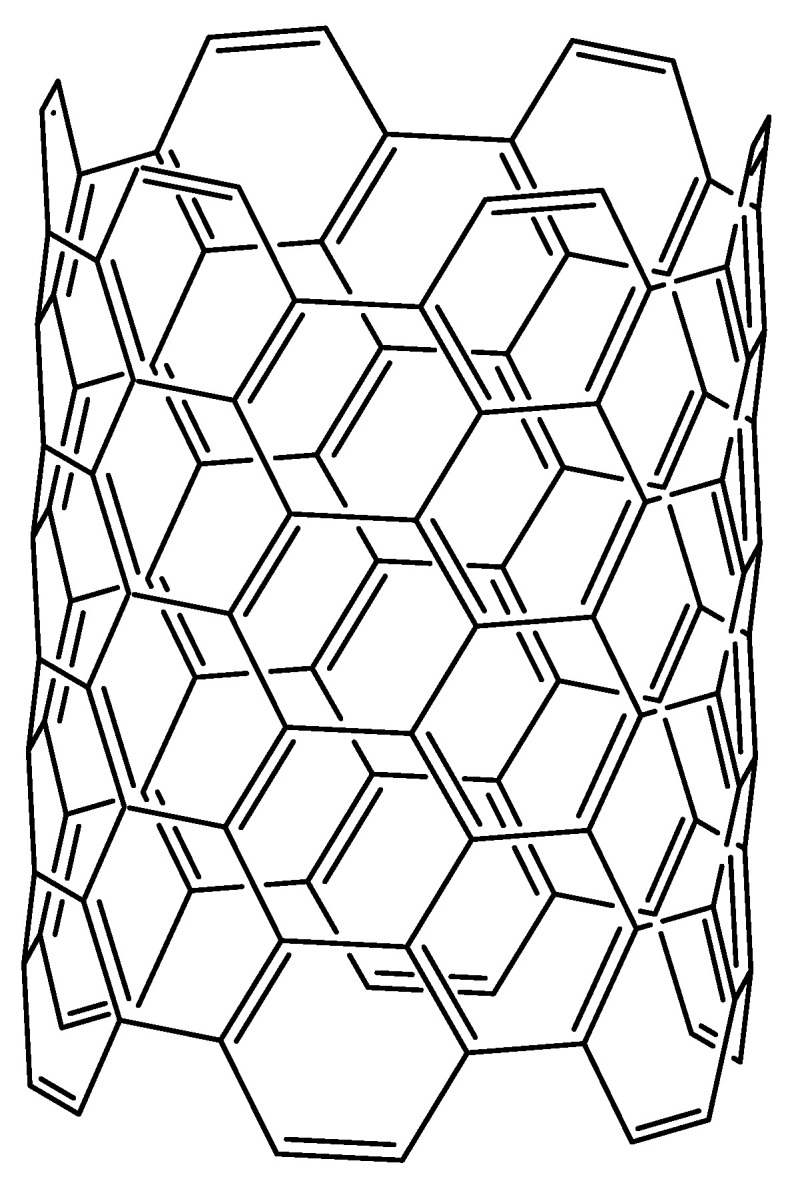
Structural representation of single-walled carbon nanotube.

Carbon nanotubes are a classic example of functional reinforcement material for construction purposes. They are one-atom thin sheets of graphite shaped into a hollow cylinder [52] as can be seen in Figure 5. Although carbon nanotubes (CNTs) are considered human engineered materials, carbon nanostructures have been found in ice cores and oil wells, thus fostering the premise that nature can provide appropriate conditions for CNT synthesis [145]. CNT’s properties such as good tensile stability, high Young’s modulus and high surface density make it ideal for use in bitumen. Their carbon nature also means they have a high chemical compatibility with bitumen. The use of CNTs as additives for bituminous mixes produces asphalt with enhanced water, shear, cold and heat resistance and also increased strength making CNT’s an excellent bitumen additive [146,147]. Saeed et al. [148] investigated the proportion in which CNT’s should be added to bitumen to give the best performance, and their findings revealed that CNTs, when added in a proportion 1.2% by weight of bitumen, produce the best results. The initial economic cost of using carbon nanotubes as bitumen modifiers compared to that of the other types of additives is higher, but the total net cost or road pavement construction and maintenance is significantly less as material consumption decreases [149].

## 6. Other Waste-Derived Biomaterials

Waste-derived materials are a principal source of degradable biomaterials which have diverse applications. In this review, a few waste-derived biomaterials have already been discussed under other different biomaterial categories. However, a few of these waste materials which confer beneficial properties on bituminous mixes do not directly fall into any of the other biomaterial categories mentioned earlier. These waste materials include industrial waste products or domestic and urban waste which are processed in ways different from conventional methods.

A study carried out by Perez et al. [21] involved the use of a liquid industrial waste rich in lignin biopolymer as a bitumen extender for asphalt mixes. They tested this waste material as a partial substitute for bitumen in asphalt mix by combining it in different percentages with bitumen to determine which proportion of bitumen–waste mixture is capable of performing as a high quality, performant asphalt mix. Their findings demonstrated that asphalt mixtures containing 20% industrial waste performed optimally and that the industrial waste is ideal for use as a bitumen extender in asphalt mixtures. The lignin-rich industrial waste was shown to improve the moisture damage resistance, thermal damage resistance and bitumen-aggregate adhesion of the asphalt mix. It is also worth noting that the industrial waste used did not undergo any transformation process and was used as is. This represents a huge step towards achieving sustainable development and a circular economy in addition to being an effective resource conservation initiative. Another study carried out by Caputo et al. [150] showed how the organic fraction of urban domestic waste subjected to the FENTON process can be used as an additive for bituminous mixes. The organic domestic waste which was mainly composed of cellulose, fatty acids and medium molecular weight hydrocarbons was treated with ferrous sulphate and hydrogen peroxide solution and was found to improve some of bitumen’s properties. Their results showed that this organic waste-derived additive at a dosage of 2% by weight of binder can be used as a filler and as a viscosifying agent for bitumen. Another eco-friendly initiative was presented by the study carried out by Calandra et al. [151] in which they evaluated the effect of waste from mining processes on the performance of bituminous mixes. The waste product (mining tailings) from manganese ore mining processes was processed into powder form and used as a filler-type additive for bitumen at dosages of 1, 3, 5 and 10% (*w*/*w*). The findings of the study indicated that bituminous mixes containing the additive at 10% dosage has a higher resistance to stress, rutting and fatigue and even resistance to aging. The pavements obtained from these mixes also has a superior durability. The effectiveness of the filler was a consequence of the favorable polar interactions between the asphaltenes and resins of bitumen and the inorganic particulate surface via the hydroxyl groups of the oxides and hydroxides of the metal oxides of the mining waste. A wider application of these waste recycling and reuse initiatives in industrial processes will go a long way in facilitating green, eco-friendly practices in the asphalt industry.

## 7. Conclusions

Research in the area of biomaterial technology is moving at a fast pace and with time, new functional biodegradable materials are being brought to light and investigated for their practicality and their potential to mitigate the looming environmental and economic crises in today’s world. Scientific breakthroughs in this field are important in the bid to create a sustainable circular economy alongside technological advancement, and biomaterials can play a key role in achieving this. With the asphalt industry being also a contributing body to the emission of toxic fumes and chemicals, which pose a threat to environmental safety and human health, the depletion of non-renewable resources and high energy consumption, eco-friendly initiatives are being put in place in order to facilitate processes in the asphalt industry that pose no threat to the environment. This review sought to highlight developments in research that can amplify the use of biodegradable materials in the production of bituminous mixes for asphalt pavements. This review draws attention to the different types of biomaterials which have the potential to be used as bio-asphalt binders either in part or entirely. The findings from the research studies highlighted herein show the potential of these materials, possible drawbacks and things to be improved upon while also giving recommendations on further steps to be taken in perfecting the effective use of these biomaterials in asphalt. Specific talking points based on the findings of the research studies highlighted in this review are:

- Bio-oils obtained are in general very useful biodegradable surfactants and asphalt additives due to their ability to induce oxypolymerization reactions via crosslinking of their structural units in bituminous mixes, thereby increasing the tensile strength of the mixes.
- Biopolymers occur in different forms, with polysaccharides being the most common source of biomaterials in this category. Polysaccharides are already widely used in the asphalt industry and are very functional in improving bitumen in various ways depending on the chemical composition of the polysaccharide. Bioelastomers such as natural rubber are also very good biomaterial additives for bituminous mixes due to crystallization phenomenon of cooled rubber in asphalt.
- Natural waxes are viscosity modifiers which enhance workability of asphalt mixes and in some cases improve adhesion between bitumen and aggregates.
- Nanomaterials from natural sources are excellent asphalt modifiers due to their ability to form nanostructures within the asphalt conglomerate.
- Waste-derived materials, apart from promoting recycling in a very utilitarian way, also bring about the improvement of the bitumen’s mechanical properties.

## Figures and Tables

**Table 1:** Elemental and substance composition of pyrolysis oils derived from biomass. (Table adapted from Oasmaa et al. [41].

Component	Bio-Oil % Composition
Carbon (wt%)	50–60
Hydrogen (wt%)	6–7
Oxygen (wt%)	35–40
Nitrogen (wt%)	<0.4
Sulphur (wt%)	<0.05
Water (wt%)	20–30
Solids (wt%)	0.01–0.1
Ash (wt%)	0.01–0.2

## Data Availability

Not applicable.
